# A Predictive Model Based on the Gut Microbiota Improves the Diagnostic Effect in Patients With Cholangiocarcinoma

**DOI:** 10.3389/fcimb.2021.751795

**Published:** 2021-11-23

**Authors:** Tan Zhang, Sina Zhang, Chen Jin, Zixia Lin, Tuo Deng, Xiaozai Xie, Liming Deng, Xueyan Li, Jun Ma, Xiwei Ding, Yaming Liu, Yunfeng Shan, Zhengping Yu, Yi Wang, Gang Chen, Jialiang Li

**Affiliations:** ^1^ Department of Hepatobiliary Surgery, The First Affiliated Hospital of Wenzhou Medical University, Wenzhou, China; ^2^ Key Laboratory of Diagnosis and Treatment of Severe Hepato-Pancreatic Diseases of Zhejiang Province, The First Affiliated Hospital of Wenzhou Medical University, Wenzhou, China; ^3^ Department of Epidemiology and Biostatistics, School of Public Health and Management, Wenzhou Medical University, Wenzhou, China; ^4^ Department of Gastroenterology, The Affiliated Drum Tower Hospital of Nanjing University Medical School, Nanjing, China; ^5^ Department of Gastroenterology, Zhongshan Hospital Xiamen University, Xiamen, China

**Keywords:** cholangiocarcinoma, gut microbiome, non-invasive diagnosis, malignant obstructive jaundice, BMI

## Abstract

Cholangiocarcinoma (CCA) is a malignant hepatic tumor with a poor prognosis, which needs early diagnosis urgently. The gut microbiota has been shown to play a crucial role in the progression of liver cancer. Here, we explored a gut microbiota model covering genera *Burkholderia-Caballeronia-Paraburkholderia*, *Faecalibacterium*, and *Ruminococcus_1* (B-F-R) for CCA early diagnosis. A case-control study was conducted to enroll 53 CCA patients, 47 cholelithiasis patients, and 40 healthy controls. The feces samples and clinical information of participants were collected in the same period. The gut microbiota and its diversity of individuals were accessed with 16S rDNA sequencing, and the gut microbiota profile was evaluated according to microbiota diversity. Finally, four enriched genera in the CCA group (genera *Bacteroides*, *Muribaculaceae_unclassified*, *Muribaculum*, and *Alistipes*) and eight enriched genera in the cholelithiasis group (genera *Bifidobacterium*, *Streptococcus*, *Agathobacter*, *Ruminococcus_gnavus_group*, *Faecalibacterium*, *Subdoligranulum*, *Collinsella*, *Escherichia-Shigella*) constitute an overall different microbial community composition (P = 0.001). The B-F-R genera model with better diagnostic value than carbohydrate antigen 19-9 (CA19-9) was identified by random forest and Statistical Analysis of Metagenomic Profiles (STAMP) to distinguish CCA patients from healthy controls [area under the curve (AUC) = 0.973, 95% CI = 0.932–1.0]. Moreover, the correlative analysis found that genera *Burkholderia-Caballeronia-Paraburkholderia* were positively correlated with body mass index (BMI). The significantly different microbiomes between cholelithiasis and CCA were found *via* principal coordinates analysis (PCoA) and linear discriminant analysis effect size (LEfSe), and Venn diagram and LEfSe were utilized to identify four genera by comparing microbial compositions among patients with malignant obstructive jaundice (MOJ-Y) or not (MOJ-N). In brief, our findings suggest that gut microbiota vary from benign and malignant hepatobiliary diseases to healthy people and provide evidence supporting gut microbiota to be a non-invasive biomarker for the early diagnosis of CCA.

## Introduction

Cholangiocarcinoma (CCA) is the second most common hepatic malignant tumor with a constantly rising morbidity in the last four decades (Saha et al., 2016; Rizvi et al., 2018). Surgical resection and biopsy remain the primary treatment and diagnosis options for CCA patients. However, due to the difficulty of access to anatomic location, highly desmoplastic, pauci-cellular nature, and asymptomatic in the early stage of CCA, it is usually diagnosed with late stage during patient’s first visit (Jarnagin et al., 2001; Sinakos et al., 2011; Barr Fritcher et al., 2015; Gonda et al., 2017; Macias et al., 2018). There are many novel diagnostic biomarkers for CCA, such as carbohydrate antigen 19-9 (CA19-9), carcinoembryonic antigen (CEA), and imaging features, whereas the diagnosis efficiency of those approaches is still unsatisfactory (Sinakos et al., 2011; Liang et al., 2015; Chen et al., 2018; Silsirivanit et al., 2020). Hence, developing a high-sensitivity and -specificity non-invasive tool for CCA early diagnosis is urgently needed.

The intestinal microbiota is a critical environmental factor for the development of liver diseases through the gut–liver axis (Sabino et al., 2016; Kummen et al., 2017; Adolph et al., 2018; Tang et al., 2018; Tripathi et al., 2018). The gut microbiome composition has been observed to differ among diseases and has also been implicated in the occurrence and progression of tumor including but not limited to liver cancer (Schwabe and Greten, 2020). The inflammatory cancer-promoting microenvironment is a factor that participates in regulating the severity of liver disease (Dapito et al., 2012; Zhang et al., 2012); meanwhile, the inflammatory signals emerging from an altered gut microbiome have been considered a new potential carcinogenic mechanism (Darnaud et al., 2013). Few studies have investigated the intestinal microbiota profile of CCA patients (Kendall et al., 2019), and it is not easy to distinguish the distribution of intestinal microbiota in individuals with CCA from people with other liver diseases (D’Amico, 2013; Chassaing et al., 2014). Hence, the diagnostic potential of microbiota for CCA remains to be revealed.

In this study, we conducted a case-control study to identify the intestinal microbiota of 40 healthy control, 53 CCA patients, and 47 cholelithiasis patients [cancer-free (CF)] using the 16S rDNA sequencing. We revealed and analyzed the differences in the microbial spectrum of control and CCA groups and put forward a specific gut microbiome composition for CCA early diagnosis. Furthermore, we characterize the intestinal microbiome of CCA patients with malignant obstructive jaundice (MOJ). In short, we defined a B-F-R genera model that covers genera *Burkholderia-Caballeronia-Paraburkholderia*, *Faecalibacterium*, and *Ruminococcus_1* as a potential non-invasive biomarker to distinguish CCA patients from healthy people and provide a new option for clinical diagnosis.

## Materials and Methods

### Participant Recruitment

Forty healthy individuals, 47 patients diagnosed with cholelithiasis, and 53 patients with primary CCA were recruited from December 2018 to October 2020 at the First Affiliated Hospital of Wenzhou Medical University. CCA patients were diagnosed according to the National Comprehensive Cancer Network (NCCN) guidelines (Benson et al., 2017) and histologically confirmed. The criteria for excluding participants were as follows: (1) ≤18 years old; (2) history of other malignancies; (3) receiving any chemoradiation, interventional or immunological therapy; (4) receiving antibiotics or probiotics therapy within the latest 8 weeks; (5) inflammatory bowel disease, symptoms of gastrointestinal obstruction, or bacterial diarrhea within the lastest 6 months. Participants’ comprehensive baseline demographic and clinicopathological information was collected, including age, gender, body mass index (BMI), smoking and alcohol habits, history of cirrhosis, hepatitis B virus (HBV) infection history, and serum tumor markers. The study complies with national standards for ethical, legal, and regulatory requirements and abides by the 2008 Helsinki Declaration and its amendments. The sample collection was in accordance with medical confidentiality and standard procedures. All respondents signed an informed consent, and the study protocol was approved by the Ethics Committee of the First Affiliated Hospital of Wenzhou Medical University (Ref No. 2020-074).

### Sample Collection, DNA Extraction, and 16S rDNA Sequencing

All fecal samples from participants were freshly collected before treatment during the hospital stay and immediately frozen and stored in a −40°C freezer within 3 h of sampling (Wei et al., 2020). To reduce the effect of sampling bias, the middle portion of fecal matter was sampled in all cases. Bacterial genomic DNA was extracted using the EZNA^®^ Stool DNA Kit (D4015, Omega, Inc., USA). The V3–V4 region of prokaryotic (bacterial and archaeal) small-subunit (16S) rDNA was amplified using slightly modified versions of the primers: 341F (5ʹ-CCTAGGGNGGCWGCAG-3ʹ) and 805R (5ʹ-GACTACHVGGGTATCTAATCC-3ʹ). The DNA extraction process was carried out in ultrapure water to exclude the possibility of false-positive PCR results. The PCR products were purified by AMPure XT beads (Beckman Coulter Genomics, USA) and quantified using Qubit (Invitrogen, USA). The amplicon pools were prepared for sequencing using the Agilent 2100 Bioanalyzer (Agilent, USA), and Illumina’s Library Quantification Kit (kappa Bioscience, USA) was used to evaluate the size and number of amplified sub-libraries. Samples were sequenced on an Illumina NovaSeq platform according to the manufacturer’s recommendations, and the sequencing service was provided by LC-Bio Technology Co., Ltd., China.

### Bioinformatic Analysis

Raw reads were analyzed using QIIME2 software. Quality filtering of the raw reads was performed using specific filtering conditions in fqtrim software (V.0.9.4) to obtain high-quality clean tags. Sequences with ≥100% similarity were assigned to the same feature. The DADA2 software was used to filter the sequencing reads and construct the feature table and sequences. As a result, the average reads were 63,191 (min = 39,815, max = 89,043). Sequence alignment for species annotation was performed using BLAST, and the alignment database used was SILVA and NT-16S. Analysis of the dominant species in different groups and multiple sequence alignment was conducted using the MAFFT software (V.7.310). Alpha diversity of samples was described by the Chao1, observing species, goods_Coverage, Shannon, and Simpson indexes, which were calculated using Qiime2(2019.7), and P value was counted by Wilcoxon test. Beta diversity was calculated by principal coordinates analysis (PCoA) using R *ade4* and *vegan* package.

### Statistical Analysis

The continuous variables were presented as mean ± standard deviation or median and interquartile range (IQR), and the categorical variables were presented as frequency (percentages). Mann–Whitney test was performed for difference analysis of non-normal distribution; chi-square test was performed for difference analysis of two groups of classification variables. Wilcoxon rank-sum test was performed to identify significant differences in microorganism abundance using Statistical Analysis of Metagenomic Profiles (STAMP; v2.1.3), and linear discriminant analysis (LDA) effect size (LEfSe) analysis (https://huttenhower.sph.harvard.edu/galaxy/) was used to determine the differently enriched microorganisms. The random forest algorithm was applied to elucidate the influence of clinical variables and microorganisms on the CCA and cholelithiasis. Further analyses were carried out in R software (v3.5.2). The receiver operating characteristic (ROC) curve of the B-F-R model was established using the R *pROC* package. To validate the stability of the model, individuals in the cohort were randomly assigned into the training cohort or validation cohort by a halfling. In the construction of the diagnostic model, data of the training group were used, and a validation cohort was used for internal validation. Ten-fold cross-validation followed by ROC curve was used to validate and show the prediction performance of the B-F-R model. The area under the curve (AUC) of the B-F-R model was calculated to evaluate the performance of this potential biomarker. The correlations between clinical variables and microorganisms were analyzed and displayed by a heatmap. P < 0.05 was considered statistically significant.

## Results

### Demographic and Clinicopathological Characteristics of the Participants

All participants in our cohort were negative for hepatitis C virus (HCV). Moreover, 21 (39.6%) of 53 CCA patients were accompanied by MOJ, which is consistent with the epidemiological characteristics of CCA in the last two decades (Jia et al., 2020). A total of 23 (43.3%) of 53 CCA patients were classified as early stage (TNM stages I–II). The median levels of CA19-9, CEA, and alpha fetoprotein (AFP) were 326.2 μg/L, 3.2 ng/ml, and 3.0 μg/L, respectively. The detailed characteristics of the participants are presented in **Table 1**.

**Table 1:** The demographic and clinicopathological characteristics of the participants.

Characteristics	Healthy control (n = 40)	CCA patients (n = 53)	Cholelithiasis patients CF (n = 47)	P value^†^
Demographics				
Age (years), median (IQR)	53.6 (43,64.5)	67.1 (61.5,73.0)	53.0 (44.5,66.0)	<0.001^MW^
Gender (male/female)	8/32	32/21	22/25	<0.001^CS^
BMI (kg/m^2^), median (IQR)	23.6 (43,64.0)	22.0 (19.4,24.6)	23.7 (21.6,25.2)	0.025^MW^
Smoking history, n (%)	6 (15.0)	15 (28.3)	7 (14.8)	0.129^CS^
Drinking history, n (%)	8 (20.0)	16 (30.1)	6 (12.7)	0.266^CS^
Dietary habit	Mixed diet	Mixed diet	Mixed diet	—
Hepatic disease history, n (%)				
Cirrhosis	0 (0.0)	6 (11.3)	0 (0.0)	0.035^F^
HBV-infected	2 (5.0)	16 (28.3)	7 (14.8)	0.005^CS^
AFP, μg/L	—	3.0 (2.2,4.3)		—
Tumor marker, median (IQR)				
CEA, ng/ml	—	3.2 (2.5, 12.8)		—
CA19-9, μg/L	—	326.2 (37.7, 1,502.1)	—	—
TNM, stages I–II, n (%)	—	23 (43.3)	—	—

^†^P value was compared between CCA patients and healthy control. Statistical methods annotation in the table: CS, chi-square test; F, Fisher’s exact test; MW, Mann–Whitney test.

AFP, alpha fetoprotein; BMI, body mass index; CA19-9, carbohydrate antigen 19-9; CCA, cholangiocarcinoma; CEA, carcinoembryonic antigen; HBV, hepatitis B virus; IQR, interquartile range.

### Gut Microbiome Features of Cholangiocarcinoma, Cholelithiasis, and Healthy Control Individuals

**Figure 1:**
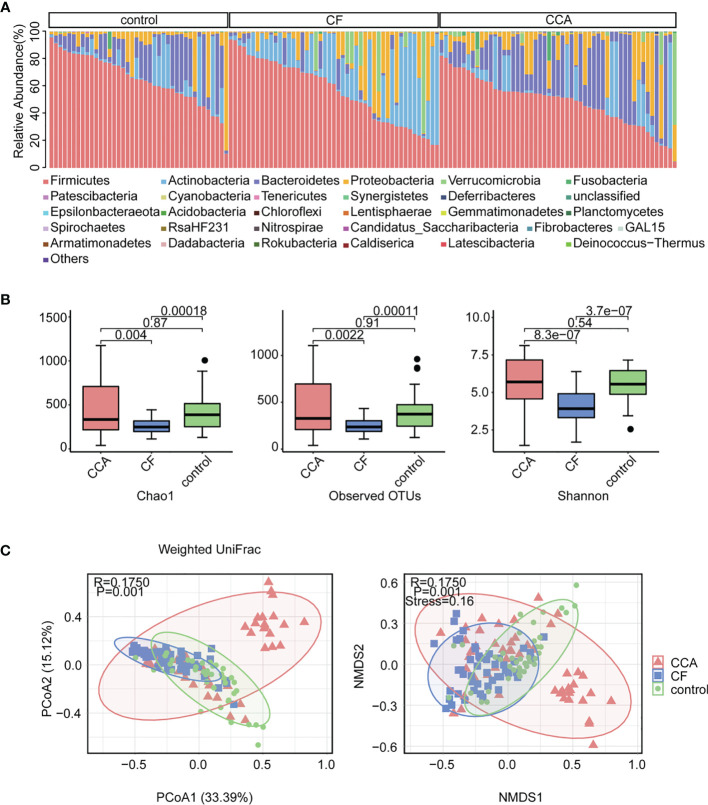
Abundance and biodiversity of gut microbiota in control (n = 40), cholelithiasis [cancer-free (CF)] (n = 47), and cholangiocarcinoma (CCA) (n = 53). **(A)** Relative abundance comparisons of the dominant bacteria in phylum level among control, CF, and CCA. **(B)** Chao1, operational taxonomic units (OTUs), and Shannon diversity index in the three cohorts were shown with box plot. The box represented the interquartile range, and the midline in the box represented the median. **(C)** Non-metric multidimensional scaling (NMDS) index and principal coordinates analysis (PCoA) based on weighted UniFrac distance metric of control, CF, and CCA (P = 0.001, P = 0.001, respectively).

Based on the adequate 16S rDNA sequences (**Figure S1**), we got 8,088,507 features among fecal samples of all participating individuals. Venn diagrams among the three groups indicated that two phyla and 68 genera were present in the CCA group only, while three phyla and 66 genera were in the cholelithiasis group, and three phyla and 109 genera were in the healthy control group (**Figure S2**). According to the bar plots of phylum taxonomic levels, Phylum *Firmicutes* was the most abundant phylum in the three groups (**Figure 1A**). Alpha diversity and beta diversity were crucial indices when analyzing gut microbiota (Bajaj et al., 2014; Gunasekaran et al., 2020). CCA and healthy control participants have markedly higher Chao1, observed number, and Shannon diversity than those of cholelithiasis individuals (P < 0.01 for each index), while no difference was found between CCA and healthy control individuals (**Figure 1B**). Furthermore, a significant global difference of microbiome composition on genus levels between the three groups was confirmed by PCoA (weighted UniFrac P = 0.001, R = 0.175) (**Figure 1C**) and non-metric multidimensional scaling (NMDS) (P = 0.001, R = 0.175, Stress = 0.16) (**Figure 1C**). In short, the composition of gut microbiota alters in liver diseases and is diverse from each other.

### Gut Microbiome-Based Predictive Models for Cholangiocarcinoma Diagnosis

**Figure 2:**
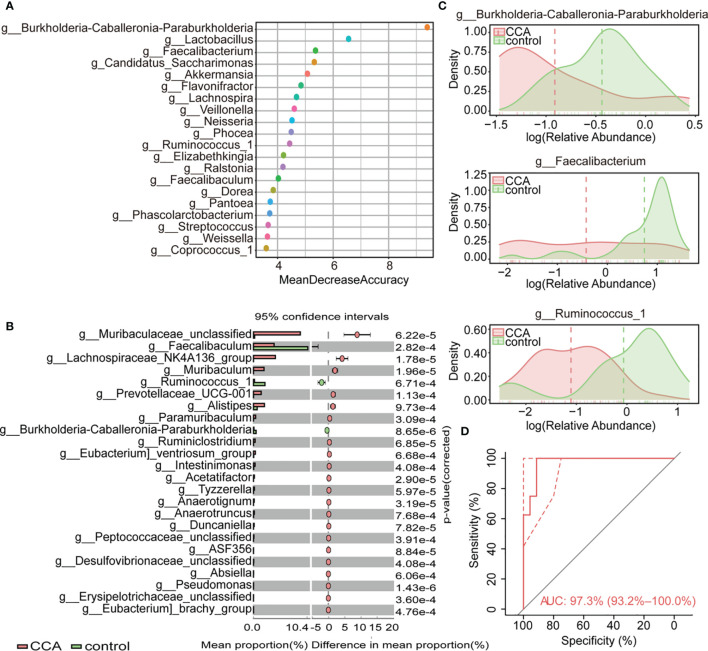
Variations of fecal microbiota composition among cholangiocarcinoma (CCA; n = 53) and control (n = 40). **(A)** The top 20 most important genera for discriminating CCA from control were screened out by random forest (RF). Each genus is ranked according to mean decrease accuracy. **(B)** Relative abundances of top 24 differentially expressed genera were evaluated by the Statistical Analysis of Metagenomic Profile (Eelch’s test, P < 0.001). The left and right panel demonstrated the average relative abundance and the 95% confidence interval of each genus in CCA and control, respectively. **(C)** Density curve of g_Burkholderia-Caballeronia-Paraburkholderia, g_Faecalibacterium, and g_Ruminococcus_1 (B-F-R) based on their relative abundances. **(D)** Classification effect of B-F-R genera model was assessed by receiver operating characteristic (ROC) curve.

To establish a gut microbiome-based predictive model for non-invasively diagnosed CCA, 24 significantly different genera (P < 0.001) among the CCA group and healthy control group were screened out using STAMP (**Figure 2A**). To further investigate which taxa contributed to the observed differences between the intestinal microbiomes of the CCA and healthy control groups, we developed random forests (RFs) with the genus-level relative abundance data to visualize the top 20 influential genera among CCA and healthy control groups. As a result, genera *Burkholderia-Caballeronia-Paraburkholderia* were the most significant, while genus *Lactobacillus* was the second important genus (**Figure 2B**). Most importantly, clinical factors that may contribute to the microbial variation were excluded, including demographics and hepatic disease history factors. There was no difference among individuals with different clinical characteristics (**Figure S3**), indicating that the disease rather than clinical features mainly explained the different microbiota composition between the two groups.

The above results provide evidence for the use of stool tests to assist the diagnosis of CCA. We crossed the data of STAMP and RF to find out the genera with the most significant difference and remarkable influence, and a B-F-R genera model including genera *Burkholderia-Caballeronia-Paraburkholderia*, *Faecalibacterium*, and *Ruminococcus_1* was identified as a candidate marker for discriminating CCA individuals from a healthy control. Density curve revalidated discrepant distribution of three biomarker genera between CCA and healthy controls (**Figure 2C**). ROC curve indicated the valid differential diagnosis ability of the B-F-R genera model with an AUC of 0.973 (95% CI = 0.932–1.0) (**Figure 2D**). In summary, our data explicated the effect of the disease itself on the different microbiota compositions between the CCA patients and healthy controls, and an efficient genera model was verified for CCA diagnosis.

### Association Between Clinical Characteristics and Gut Microbe

**Figure 3:**
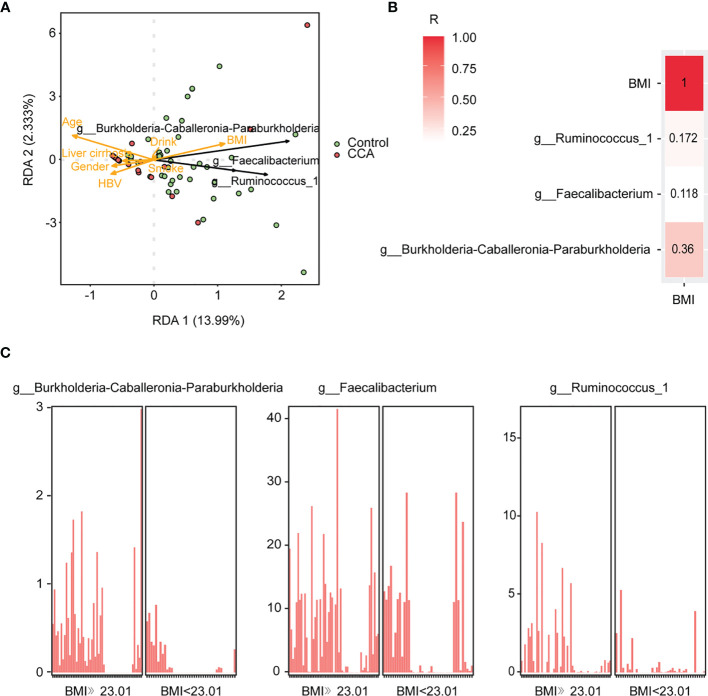
The correlation of genera and clinical variables in control and cholangiocarcinoma (CCA). **(A)** Redundancy analysis of the three genera and clinical variables. **(B)** The correlation between body mass index (BMI) and microbial markers. The higher value indicates stronger correlation. **(C)** Abundance of g_Burkholderia-Caballeronia-Paraburkholderia, g_Faecalibacterium, and g_Ruminococcus_1 in individuals with different BMIs.

Redundancy analysis (RDA) was utilized to reveal the underlying relationships between clinical characteristics and gut microbiota. The result showed that healthy individuals tended to distribute on the BMI side (**Figure 3A**). And, the genera *Burkholderia-Caballeronia-Paraburkholderia* remarkably positively correlated with BMI by correlation analysis (**Figure 3B** and **Figure S4**). The relative abundance of the three biomarker genera was further evaluated in participants with different BMIs, and the cutoff point of BMI was set at 23.01. Notably, all biomarker genera were relatively enriched in individuals with high BMI (≥23.01) (**Figure 3C**). To sum up, there was a positive correlation between BMI and genera *Burkholderia-Caballeronia-Paraburkholderia*.

### Gut Microbe Alterations in Cholelithiasis and Cholangiocarcinoma Patients

**Figure 4:**
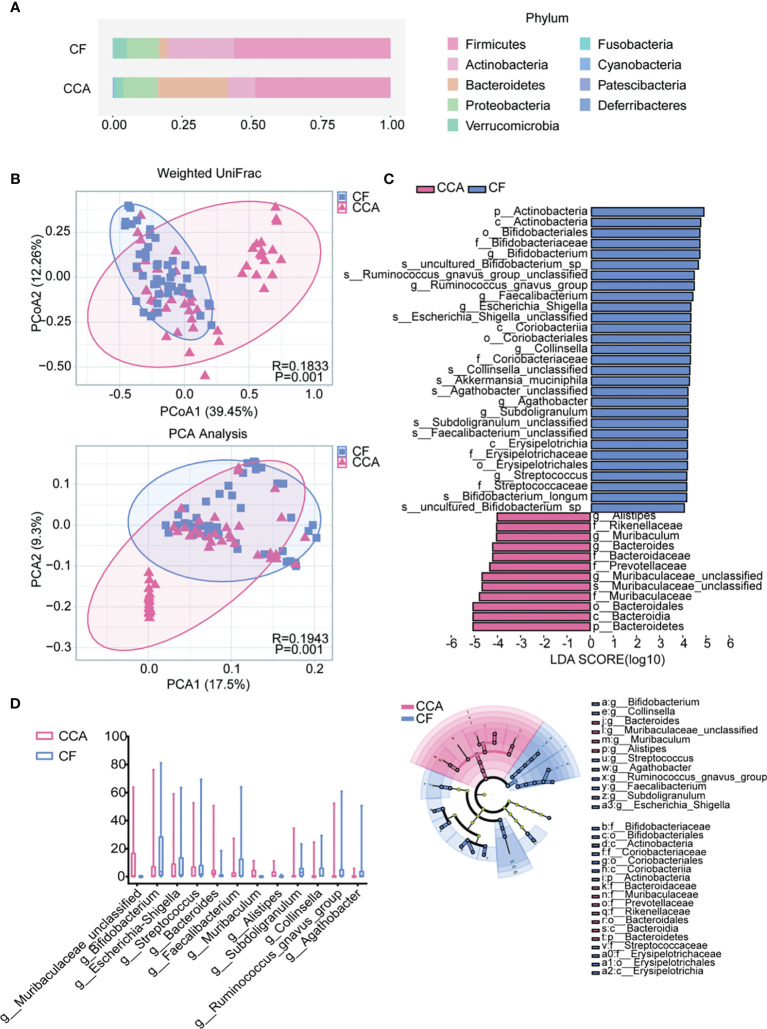
The differences in gut microbiota among the cholangiocarcinoma (CCA) and cholelithiasis [cancer-free (CF)] patients. **(A)** Composition of the gut microbiota at the phylum level. **(B)** Principal coordinates analysis (PCoA) index and principal component analysis (PCA) based on weighted UniFrac distance metric for CF and CCA (P = 0.001, P = 0.001). **(C)** Differentially abundant taxa between CF and CCA samples analyzed by linear discriminant analysis (LDA) effect size (LEfSe) were shown in histogram and cladogram. All listed taxa were significantly (Kruskal–Wallis test, P < 0.05, LDA score >4) enriched in their respective groups. **(D)** Box plot showing the relative abundances of the top 15 differentially expressed taxa identified by LEfSe (P < 0.001).

Among cholelithiasis and CCA groups, 19 phyla and 344 genera were shared among the two groups (**Figure S2**). *Firmicutes*, *Actinobacteria*, *Bacteroidetes*, *Proteobacteria*, and *Verrucomicrobia* were the five dominant phyla in cholelithiasis and CCA groups (**Figure 4A**). Based on weighted UniFrac, a significant clustering effect among the two groups was observed in PCoA and principal component analysis (PCA) (**Figure 4B**; both P = 0.001). LEfSe showed that there are 12 taxa with differentiated distribution on genus level among cholelithiasis and CCA groups (**Figure 4C**; LDA >4.0). The genera *Bacteroides*, *Muribaculaceae_unclassified*, *Muribaculum*, and *Alistipes* were significantly enriched in the CCA groups, while the genera *Bifidobacterium*, *Streptococcus*, *Agathobacter*, *Ruminococcus_gnavus_group*, *Faecalibacterium*, *Subdoligranulum*, *Collinsella*, and *Escherichia-Shigella* were significantly enriched in the cholelithiasis group (**Figure 4D**). Correlations between the different microbiomes were confirmed by the Sankey diagram (**Figure S5**). Briefly, significant differences in intestinal microbiota exist between cholelithiasis and CCA patients indeed.

### Gut Microbes Changed in Cholangiocarcinoma Patients With Malignant Obstructive Jaundice

**Figure 5:**
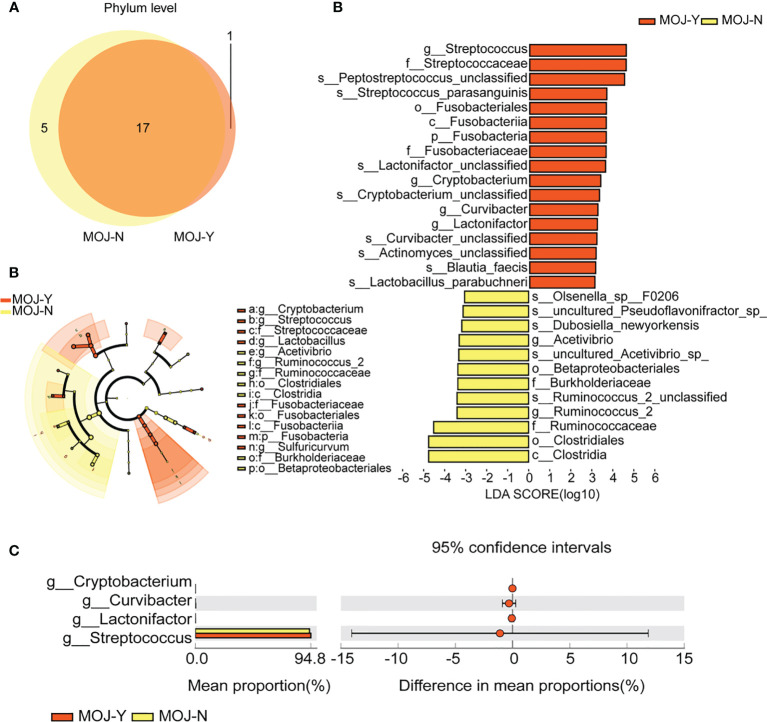
The differences of gut microbiota among patients with malignant obstructive jaundice (MOJ-Y) or not (MOJ-N). **(A)** The differential microbial species were classified using Venn diagram. **(B)** Differentially abundant taxa between MOJ-Y and MOJ-N samples analyzed by linear discriminant analysis (LDA) effect size (LEfSe) were shown in histogram and cladogram. All listed taxa were significantly (Kruskal–Wallis test, P < 0.05, LDA score >3) enriched in their respective groups. **(C)** Relative abundances of four differentially expressed genera by the Statistical Analysis of Metagenomic Profile. The left and right panels demonstrated the average relative abundance and the 95% confidence interval of each phylum in MOJ-Y and MOJ-N, respectively.

Microbial compositions among CCA patients with MOJ (MOJ-Y) or not (MOJ-N) were compared firstly using Venn diagram. Here, 17 phyla were shared among MOJ-N and MOJ-Y groups (**Figure 5A**), and phylum *Nitrospirae* was the only one present in the MOJ-Y, which indicated changes in gut microbiota among CCA patients with MOJ. Unfortunately, phylum *Nitrospirae* was eliminated in the later research, since its relative abundance in MOJ-Y group was under 1% (**Table S1**). In taxonomic analyses, LEfSe identified a higher relative abundance of *Streptococcus*, *Cryptobacterium*, *Lactonifactor*, and *Curvibacter* in the MOJ-Y group at the genus level compared with MOJ-N (**Figure 5B**), and STAMP displayed relative abundances of four genera (**Figure 5C**). In a word, the gut microbes would change in CCA patients when complicated by MOJ.

## Discussion

CCA is a silent malignant hepatic tumor with increasing morbidity, and extensive studies have been conducted to promote its early diagnosis. In this study, we investigated the intestinal microbiota characteristics of CCA patients in detail and provided a B-F-R genera model for the CCA diagnosis in its early stages. Furthermore, we defined the alterations of intestinal microbiota in the progress of hepatobiliary diseases.

The liver–microbiome axis plays a pivotal role in metabolic homeostasis and affects disease development via blood circulation and systemic innervation (Benson et al., 2017). Accumulating evidence suggested that the gut microbiome dysbiosis and low bacterial diversity would lead to several metabolic and inflammatory diseases (Konturek et al., 2018), especially hepatobiliary disease (Adolph et al., 2018; Ren et al., 2019). In this study, we delineated the microbiome composition of the intestinal microbiota using 16S rDNA sequencing in CCA, cholelithiasis, and healthy participants. The findings revealed that the fecal microbiome of CCA and healthy participants had increased species richness and homogeneity compared with cholelithiasis individuals, and there were no significant differences in alpha diversity between CCA and healthy individuals. Our results deepened the understanding of the correlation between the intestinal microbiome and CCA, which has not been well investigated before.

Bile acids (BAs) and antimicrobial molecules secreted by the liver that transport to intestine are conducive for the maintenance of intestinal bacteria homeostasis. The gut microbiota not only determines a differential extraction of nutrients and energy from the diet but also synthesizes a broad variety of metabolites, such as BA, choline, and short-chain fatty acids (SCFAs), serving as important signaling factors and energy substrates affecting liver functions eventually in turn (Di Ciaula et al., 2020). Genus *Bacteroides*, which is significantly enriched in CCA, has been reported to be associated with BAs, particularly with deoxycholic acid (DCA) recently (Petrov et al., 2019; Wan et al., 2020). *Muribaculaceae_unclassified* and *Lachnospiraceae_NK4A136_group* were dominant at the genus level of taxonomic classification. Interestingly, *Muribaculaceae* plays a major role in metabolizing carbohydrates and polyphenols (Chung et al., 2020; Chen et al., 2021), and *Lachnospiraceae_NK4A136_group* works as SCFA-producing-related bacterium that modulates inflammation (Hu et al., 2019; Wang et al., 2019). Thus, we proposed that *Bacteroides*, *Muribaculaceae_unclassified*, and *Lachnospiraceae_NK4A136_group* are the potential genera for CCA early detection.

To date, CA19-9 was the most commonly used tumor marker for CCA; nevertheless, the forecasting performance of CA19-9 remains unsatisfactory (AUC = 0.881) (Ren et al., 2019). Since the non-invasive biomarkers for the early detection and diagnosis of CCA have been an unmet need (Macias et al., 2018), we established a microbial-based model for CCA with accurate discriminative ability. In the B-F-R genera model, the genus *Faecalibacterium* is a butyrate-producing bacterium of the gut microbiota and plays an important role in a healthy gut (Iino et al., 2019) and has a protective effect on inflammatory bowel diseases (Sokol et al., 2008), colorectal cancer (Balamurugan et al., 2008), non-alcoholic fatty liver disease (Lensu et al., 2020), and liver failure (Wang et al., 2021). In addition, genus *Ruminococcaceae* can help reduce polysaccharide degradation and reverses dextran sodium sulfate to preserve intestinal barrier integrity (Wang et al., 2019). Previous studies suggested that gut microbiota was associated with body weight (Cuevas-Sierra et al., 2019), and obesity might be a risk factor for CCA progression and postoperative recurrence (Merath et al., 2019; Zhou et al., 2020; Yugawa et al., 2021). There was evidence concerning the potential role of gut microbiota and probiotic-derived extracellular vesicles (EVs) on obesity via the modulation of inflammation, metabolism, and gut permeability (Díez-Sainz et al., 2021). Although the clinical variables for CCA development were not included in our model, it was striking that genera *Burkholderia-Caballeronia-Paraburkholderia* were positively correlated with BMI, and biomarker genera had a higher abundance in individuals with BMI ≥23.01. Fortunately, our findings reached a unanimous conclusion and provided strong evidence for the potential of non-invasive fecal testing for the early diagnosis of CCA.

The intestinal barrier is a physical barrier formed by single-layer epithelial cells, preventing intestinal microbiota from shifting. Accumulating studies have linked intestinal barrier dysfunction to the occurrence of hepatobiliary cancer (Yu and Schwabe, 2017; Tripathi et al., 2018). Out of 12 genera defined in our data that differently distributed in cholelithiasis and CCA participants, genera *Bacteroides* (Bescucci et al., 2020) and *Alistipes (* Saulnier et al., 2011) were related to intestinal barrier dysfunction and intestinal inflammation. Besides, *Bacteroides* is one of the tumor lesion microbiota that correlated to fibrosis (Nejman et al., 2020; Yamamoto et al., 2021). The increase of intestinal permeability promoted the migration of bacteria and the elevation of serum lipopolysaccharide, following with an increase of inflammatory response caused by Toll-like receptor 4 (TLR4) and eventually promoting the progression of chronic liver disease and tumorigenesis (Lin et al., 1995; Dapito et al., 2012). In addition, present study has indicated a positive correlation between gut microbiota and mortality in patients with alcoholic hepatitis (Komiyama et al., 2021). All this evidence confirmed bacterial translocation is concerned with the progression of other liver diseases.

Cholelithiasis refers to calculi in cholecyst and bile duct, which is linked to the risk of CCA (Schottenfeld and Beebe-Dimmer, 2006; Khan et al., 2019). MOJ is mainly manifested as skin and mucosal jaundice, which is highly related to the increase of serum bilirubin (Qi and Yan, 2021). Bile stasis and intraductal intense concentration of BAs in patients with biliary obstruction may cause malignant transformation of hepatic epithelium and likely be accompanied by hepatic insufficiency, malnutrition, and hypoproteinemia, which lead to a series of complications (Kobayashi et al., 1999; Huang et al., 2017), and MOJ is a risk factor for the prognosis of patients with CCA (Cui et al., 2017). Gut microbiome is a critical factor of intestinal inflammation and modulates the metabolism and immune function of the host (Yao et al., 2021). Our results showed that four phyla were more abundant in patients with CCA complicated by obstructive jaundice. Among the differential phyla, *Streptococcus* is related to chronic alcoholic fatty liver disease, HBV-related acute-on-chronic liver failure, primary sclerosing cholangitis, etc. (Jain et al., 2021; Kang et al., 2021; Tierney et al., 2021; Yao et al., 2021) In the meantime, the infection rate of *Streptococcus* is closely related to nutrient deficiency (Guevara et al., 2020). Our result proposed the potential relationship between the CCA, MOJ, and intestinal microbiota and suggested the value and influence of intestinal microbiota on CCA early warning.

There are still a few limitations to this study. First, this is a single-center study with limited sample size. A larger multicenter trial should be conducted to validate our findings. Secondly, 16S rDNA sequencing was incapable of uncovering the full genetic contents as metagenomic sequencing does. Then, we simply treated dietary factor as control covariates by ruling out subjects with special dietary habits, such as vegetarianism or any other restriction. More samples are required in future studies to compensate for the problem. At last, the relationship between CCA subtypes and intestinal microbes remains to be discovered. Nonetheless, our study provides novel insights into the mechanisms of gut microbiome-affected disease. Our findings suggest that gut microbiota varies from benign and malignant hepatobiliary diseases to healthy people. The successful B-F-R genera model indicates the possibility of using intestinal microorganisms as non-invasive biomarkers for the early diagnosis of CCA and provides a new potential method for clinical diagnosis.

## Data Availability Statement

The original contributions presented in the study are publicly available. These data can be found here: National Center for Biotechnology Information (NCBI) BioProject database under accession number PRJNA765184.

## Ethics Statement

Written informed consent was obtained from the individuals for the publication of any potentially identifiable images or data included in this article.

## Author Contributions

JL, GC, and YW conceptualized and designed the study. TZ, SZ, CJ, ZL, XX, LD, JM, and XL collected the data and performed the analysis. All authors interpreted the data. TZ and JL drafted the initial version of the article. XD and YL offered professional suggestions and critical revisions of the article. All authors critically reviewed numerous revisions of the article and contributed important intellectual content. JL, GC, and YW had complete access to all the study data and were responsible for data integrity, the accuracy of the analyses, and the final decision to submit the article for publication. All authors contributed to the article and approved the submitted version.

## Funding

This study was supported by grants from the National Natural Science Foundation of China (81772628, 82072685, 81703310) and Provinces and Ministries Co-Contribution of Zhejiang, China (No.wkj-zj-1706). The funding was used for collection, analysis, and interpretation of data.

## Conflict of Interest

The authors declare that the research was conducted in the absence of any commercial or financial relationships that could be construed as a potential conflict of interest.

## Publisher’s Note

All claims expressed in this article are solely those of the authors and do not necessarily represent those of their affiliated organizations, or those of the publisher, the editors and the reviewers. Any product that may be evaluated in this article, or claim that may be made by its manufacturer, is not guaranteed or endorsed by the publisher.

## Abbreviations

B-F-R, *Burkholderia-Caballeronia-Paraburkholderia, Faecalibacterium* and *Ruminococcus_1*; CCA, cholangiocarcinoma; CF, cancer-free; 16S rDNA, 16S ribosomal deoxyribonucleic acid; PCoA, principal coordinates analysis; LDA, linear discriminant analysis; LEfSe, linear discriminant analysis effect size; BMI, body mass index; MOJ, malignant obstructive jaundice; AFP, alpha fetoprotein; CEA, carcinoembryonic antigen; CA19-9, carbohydrate antigen 19-9; HCV, hepatitis C virus; HBV, hepatitis B virus; TNM, Tumor Node Metastasis; RF, random forest; STAMP, Statistical Analysis of Metagenomic Profiles; NMDS, non-metric multidimensional scaling; ROC, receiver operating characteristic; AUC, area under the curve; RDA, redundancy analysis; PCA, principal component analysis; BAs, bile acids; DCA, deoxycholic acid; SCFAs, short-chain fatty acids; EVs, extracellular vesicles; TLR4, Toll-like receptor 4; OTUs, operational taxonomic units; IQR, interquartile range.
