# Supplementary material for: A Predictive Model Based on the Gut Microbiota Improves the Diagnostic Effect in Patients With Cholangiocarcinoma
Source: Front Cell Infect Microbiol. 2021 Nov 23;11:751795. doi: 10.3389/fcimb.2021.751795 (PMC8650695; doi:10.3389/fcimb.2021.751795)
Supplement: Supplementary file 1 [file DataSheet_1.docx]

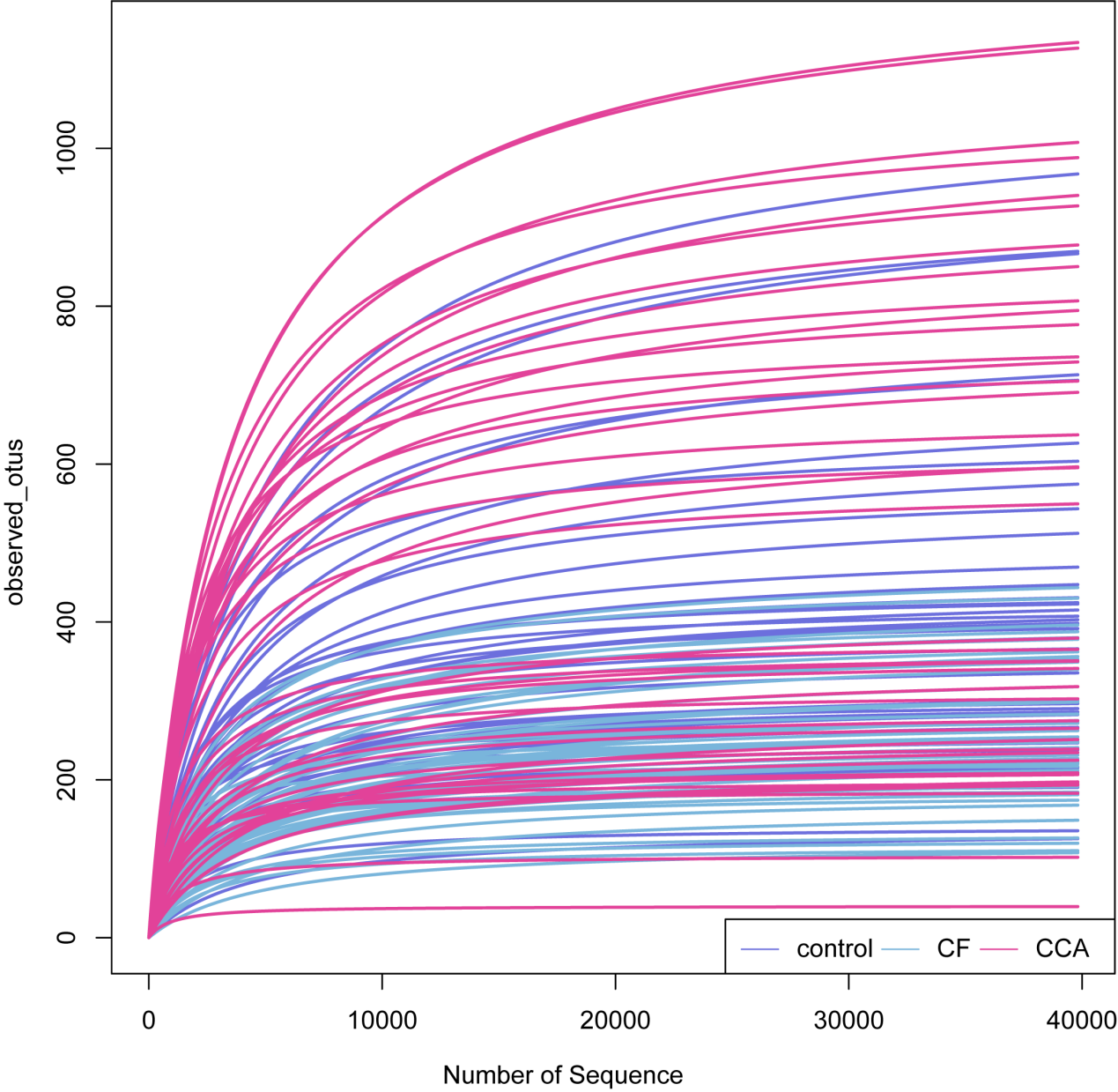


**Figure S1. The rarefaction curve reached a plateau, indicating that the sequencing depth is adequate.**


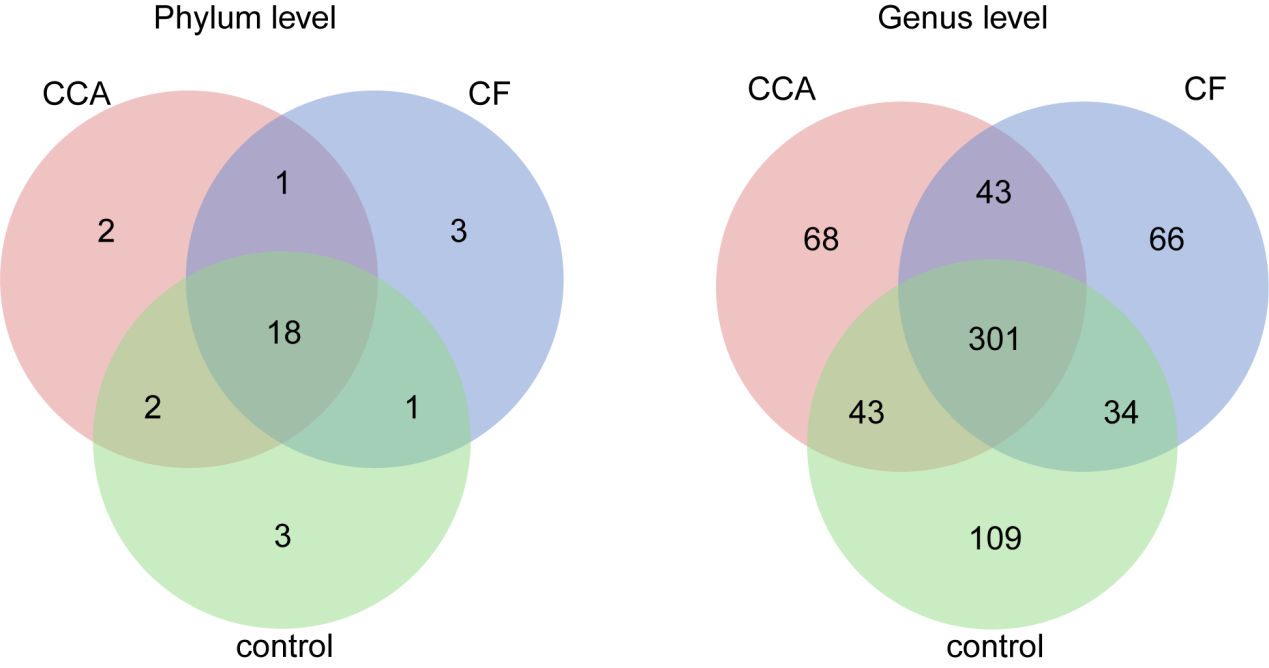


**Figure S2. Venn diagrams of gut microbiota in liver disease.**


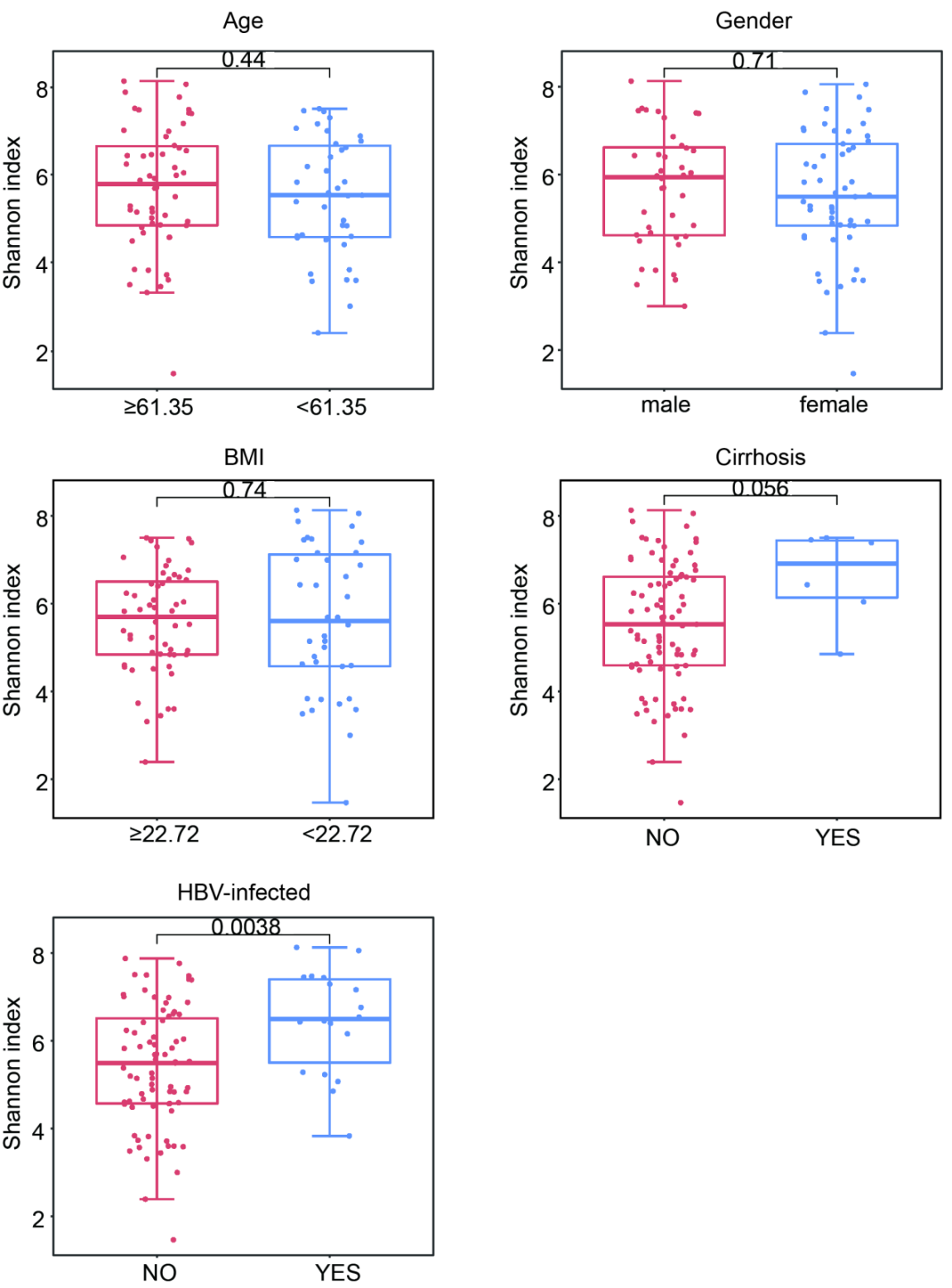


**Figure S3. The shannon index was selected to reveal difference among gut microbiota of participants and clinical variables.** Box plot revealed no difference, except for HBV infection, was found in gut microbiota of participants with different clinical varibles. The cutoff point of age and BMI index was set at 61.35 and 22.72, respectively.


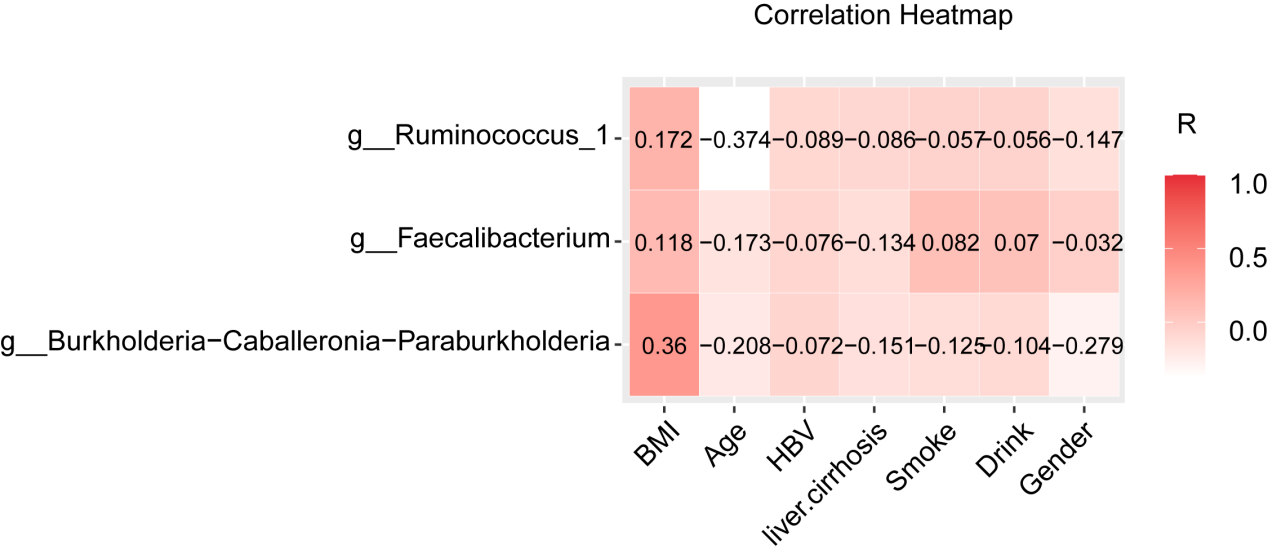


**Figure S4. Correlation coefficient heat map manifested the relationship between three genera and clinical variables.**


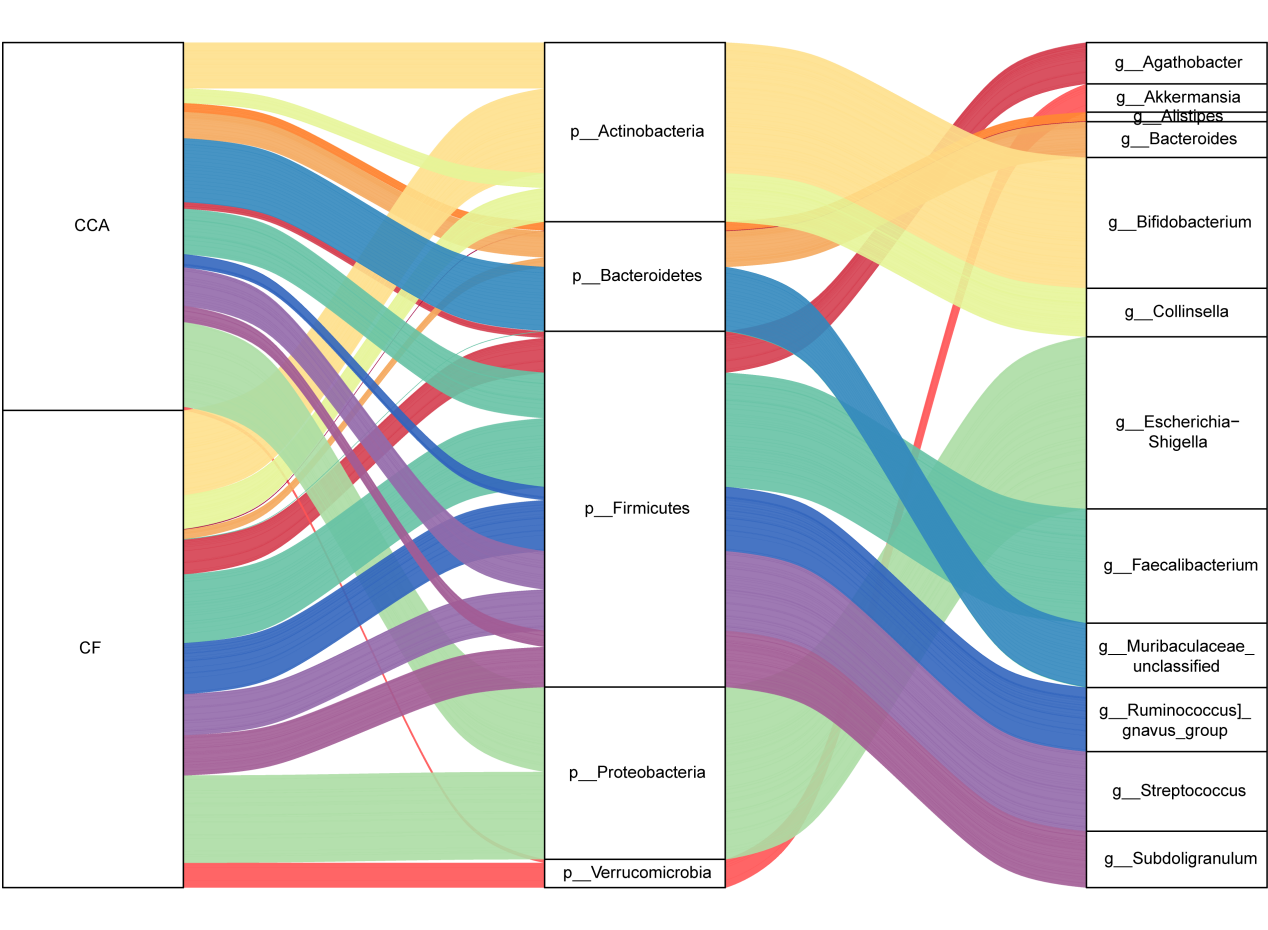


**Figure S5. Sankey diagram was applied to describe correlations between significantly different microbiomes.**

Table S1: Distribution of gut microbiota at phyla among MOJ-Y and MOJ-N.

| Phyla | Relative abundance | |
| --- | --- | --- |
|  | MOJ-Y | MOJ—N |
| p__Firmicutes | 913.51 | 1665.99 |
| p__Bacteroidetes | 419.44 | 920.54 |
| p__Proteobacteria | 347.20 | 317.74 |
| p__Actinobacteria | 301.78 | 214.68 |
| p__Verrucomicrobia | 91.18 | 42.79 |
| p__Fusobacteria | 21.32 | 22.39 |
| p__Cyanobacteria | 1.48 | 7.02 |
| p__Patescibacteria | 1.85 | 2.44 |
| p__Deferribacteres | 1.24 | 2.81 |
| p__unclassified | 0.45 | 2.32 |
| p__Synergistetes | 0.20 | 0.59 |
| p__Tenericutes | 0.04 | 0.45 |
| p__Epsilonbacteraeota | 0.22 | 0.16 |
| p__RsaHF231 | 0.04 | 0.04 |
| p__Acidobacteria | 0.02 | 0.01 |
| p__Chloroflexi | 0.00 | 0.01 |
| p__Nitrospirae | 0.01 | 0 |
| p__Spirochaetes | 0.00 | 0.00 |
| p__Lentisphaerae | 0 | 0.01 |
| p__Latescibacteria | 0 | 0.01 |
| p__Gemmatimonadetes | 0 | 0.00 |
| p__Planctomycetes | 0 | 0.00 |
| p__Candidatus_Saccharibacteria | 0 | 0.00 |
